# Cubic and Hexagonal Liquid Crystals as Drug Delivery Systems

**DOI:** 10.1155/2014/815981

**Published:** 2014-06-05

**Authors:** Yulin Chen, Ping Ma, Shuangying Gui

**Affiliations:** ^1^Department of Pharmaceutics, College of Pharmacy, Anhui University of Chinese Medicine, Hefei, Anhui 230031, China; ^2^Global Pharmaceutical Research and Development, Hospira Inc., 1776 North Centennial Drive, McPherson, KS 67460, USA; ^3^Anhui Key Laboratory of Modern Chinese Medicine & Materia, Hefei, Anhui 230031, China; ^4^Anhui “115” Xin'an Traditional Chinese Medicine Research & Development Innovation Team, Hefei, Anhui 230031, China

## Abstract

Lipids have been widely used as main constituents in various drug delivery systems, such as liposomes, solid lipid nanoparticles, nanostructured lipid carriers, and lipid-based lyotropic liquid crystals. Among them, lipid-based lyotropic liquid crystals have highly ordered, thermodynamically stable internal nanostructure, thereby offering the potential as a sustained drug release matrix. The intricate nanostructures of the cubic phase and hexagonal phase have been shown to provide diffusion controlled release of active pharmaceutical ingredients with a wide range of molecular weights and polarities. In addition, the biodegradable and biocompatible nature of lipids demonstrates the minimum toxicity and thus they are used for various routes of administration. Therefore, the research on lipid-based lyotropic liquid crystalline phases has attracted a lot of attention in recent years. This review will provide an overview of the lipids used to prepare cubic phase and hexagonal phase at physiological temperature, as well as the influencing factors on the phase transition of liquid crystals. In particular, the most current research progresses on cubic and hexagonal phases as drug delivery systems will be discussed.

## 1. Introduction

Lipid-based lyotropic liquid crystals can be mainly classified into lamellar phase (L_*α*_), cubic phase (V_2_), and hexagonal phase (H_2_) according to their different internal structures. Among them, V_2_ and H_2_ are the most important and recently have received much attention due to their highly ordered internal structures, which offers the potential as a slow release matrix for active pharmaceutical ingredients with various sizes and polarities [1, 2].

Cubic and hexagonal liquid crystals are often spontaneously formed by the addition of certain amphiphilic lipids in an aqueous environment [3]. When cubic and hexagonal liquid crystals are dispersed into nanoparticles with excess water with the addition of stabilizers (such as Pluronic copolymers [4] and Myrj series [5]), they form stable colloidal dispersions which are termed cubosomes and hexosomes, respectively [6–9]. In general, the preparation methods of cubic and hexagonal liquid crystals are easier than their dispersions. For example, cubic and hexagonal liquid crystals can be prepared by simply blending lipid and aqueous phases by vortex [10]. In contrast, the manufacture of cubosomes or hexosomes is more complicated. There are two commonly used techniques to prepare cubosomes or hexosomes. The first one is called the top-down approach. In this method, a mixture of the structure-forming lipid and stabilizers is first hydrated to allow them to self-assemble in a viscous bulk. The bulk is then dispersed into an aqueous solution through the input of high level energy (such as high-pressure homogenization [11] and ultrasonication [12]) to form cubosomes or hexosomes. The second preparation technique is called the bottom-up approach. In this approach, the key factor is the presence of hydrotropes, which can dissolve water-insoluble lipid to create liquid precursor and prevent the formation of liquid crystal at high concentration [13, 14]. Controlled addition of aqueous medium into the above mixed solution rapidly reduces the lipid solubility and thus results in the formation of cubosomes or hexosomes. This approach needs less energy input and has the advantage to produce nanoparticles with improved stability [15].

**Figure 1:**
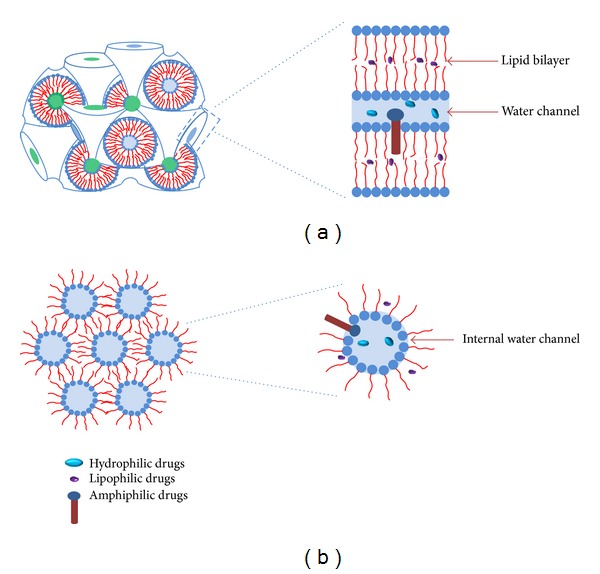
The schematic structures of (a) cubic phase (Pn3m) and (b) hexagonal phase. Possible localizations of drugs in the liquid crystals are pointed out (adapted from [19]).

The structure of cubic phase is unique and consists of two continuous but nonintersecting water channels separated by a lipid bilayer [1, 7]. Based on X-ray crystallographic studies, cubic phase is divided into three types: the double-diamond lattice cubic phase (Pn3m), the body-centered cubic lattice cubic phase (Im3m), and the gyroid lattice cubic phase (Ia3d) [2, 16]. The schematic structure of the double-diamond lattice cubic phase (Pn3m) is shown in Figure 1(a). The cubic phases are used as the carriers for hydrophilic, lipophilic, or amphiphilic drugs. Hydrophilic drugs will be located close to the polar head of lipid or in the water channels, whereas lipophilic drugs will be loaded in the lipid bilayer and amphiphilic drugs in the interface (Figure 1(a)) [17]. The hexagonal phase is composed of cylindrical micelles packed in a hexagonal pattern (Figure 1(b)). In contrast to the cubic phase, the water channels in the hexagonal phase are closed [3, 18]. The distribution of drugs in hexagonal phase is similar to that in cubic phase (Figure 1(b)).

In recent years, cubic and hexagonal phases have received considerable attention because of their potential as drug delivery systems. Cubic and hexagonal phases provide a slow drug release matrix and protect peptides, proteins, and nucleic acids from chemical and physical degradation [20–23]. The liquid crystal-forming lipids are nontoxic and biodegradable [24] and can be used for various routes of administration. In this review, we briefly introduce the lipids used to prepare cubic phase and hexagonal phase at physiological temperature, as well as the influencing factors on the phase transition of liquid crystals. In particular, the investigation of current status of cubic and hexagonal liquid crystals, along with their dispersions as drug delivery systems, will be discussed. This review is not intended to provide a comprehensive overview but focuses on lipid-based cubic and hexagonal liquid crystals and their applications in drug delivery systems.

## 2. Cubic and Hexagonal Phase-Forming Lipids

Because of their low toxic and biodegradable properties, various types of lipids have been extensively studied as the carriers to prepare lipid-based lyotropic liquid crystals. In general, lipid-based lyotropic liquid crystals are formed by swelling of certain amphiphilic lipids due to their amphiphilic nature containing a polar headgroup and a hydrophobic tail. When exposed to aqueous environment, amphiphilic lipids spontaneously form thermodynamically stable self-assembled structures and eventually develop into cubic and/or hexagonal liquid crystals depending on temperature and water content. Up until now, there have been few materials known to exhibit this kind of phase behavior. In this part, we mainly introduce the lipids which are commonly used to form cubic and/or hexagonal liquid crystals at physiological temperature.

### 2.1. Cubic Phase-Forming Lipids

Glyceryl monooleate (GMO, 2,3-dihydroxypropyl oleate) [25, 26], phytantriol (PT, 3,7,11,15-tetramethyl-1,2,3-hexadecanetriol) [27, 28], and other lipids such as monolinolein [29–31], monoelaidin [32–34], phosphatidylethanolamine [35–37], oleoylethanolamide [23], phospholipids [38–41], PEGylated phospholipids [42], alkyl glycerates [10, 43], and glycolipids [44] have been reported to form cubic phase. Among them GMO and PT are the most commonly studied to form cubic phase liquid crystals as drug delivery systems.

**Figure 2:**
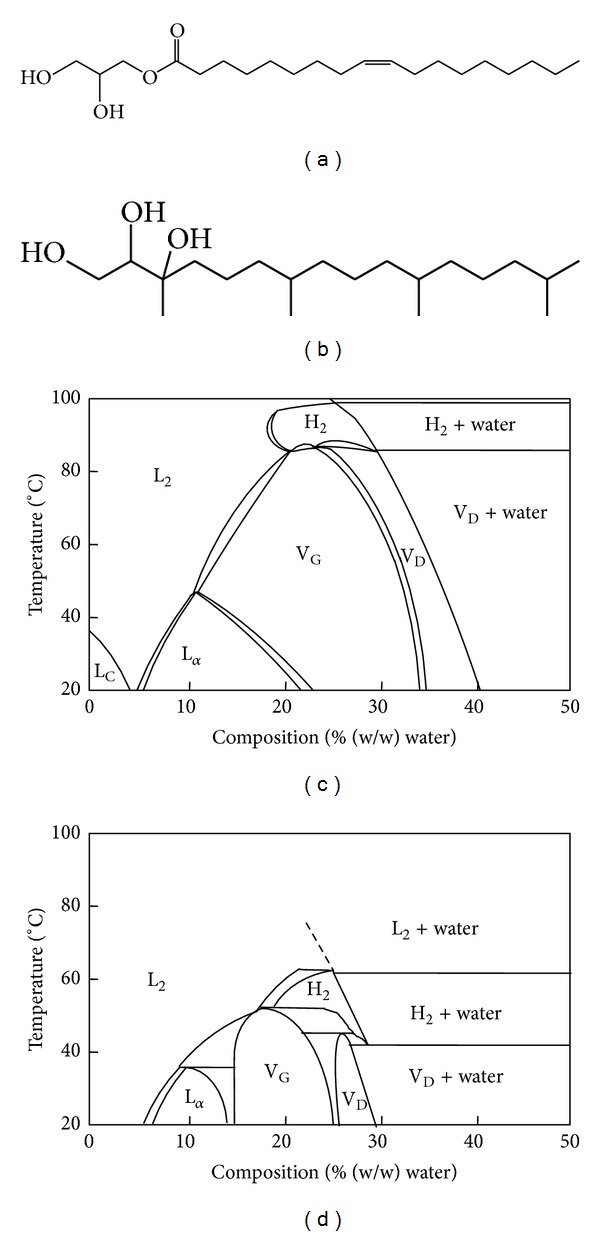
Molecular structures of (a) GMO and (b) PT. Phase behaviors of (c) GMO-water and (d) PT-water system. Phase notations: L_C_: liquid crystal; L_2_: fluid isotropic solution; L_*α*_: lamellar phase; H_2_: reversed hexagonal phase; V_G_: gyroid lattice cubic phase (Ia3d); V_D_: double-diamond lattice cubic phase (Pn3m) (adapted with modifications from [52]).

GMO, or monoolein, is a polar unsaturated monoglyceride. The chemical structure of GMO is shown in Figure 2(a). GMO is a nontoxic, biodegradable, and biocompatible material and is listed in the FDA's* Inactive Ingredients Guide.* Although GMO is a well-known emulsifying agent and food additive since the 1950s, its potential application in drug delivery has not been discovered until in 1984 that GMO was first proposed as a biocompatible material [45]. The phase diagram of GMO and water is shown in Figure 2(c). It is clearly demonstrated that GMO forms cubic phase with water in a broad area at physiological temperature. Due to its excellent phase behavior, GMO is widely used for the preparation of cubic liquid crystals at ambient temperatures.

Because of the similar phase behavior to GMO, PT also has been widely used as a cubic phase-forming material in the past decade [27, 46, 47]. The chemical structure and phase behavior of PT are illustrated in Figure 2. PT is a well-known active ingredient used in cosmetic industry, such as hair and skin care products [48]. Recently, PT has been receiving more attention since it has a phytanyl backbone but does not possess an ester bond, which provides an improved chemical stability compared to other fatty acid-based materials, such as GMO, in aqueous and model gastrointestinal conditions [49–51].

### 2.2. Hexagonal Phase-Forming Lipids

**Figure 3:**
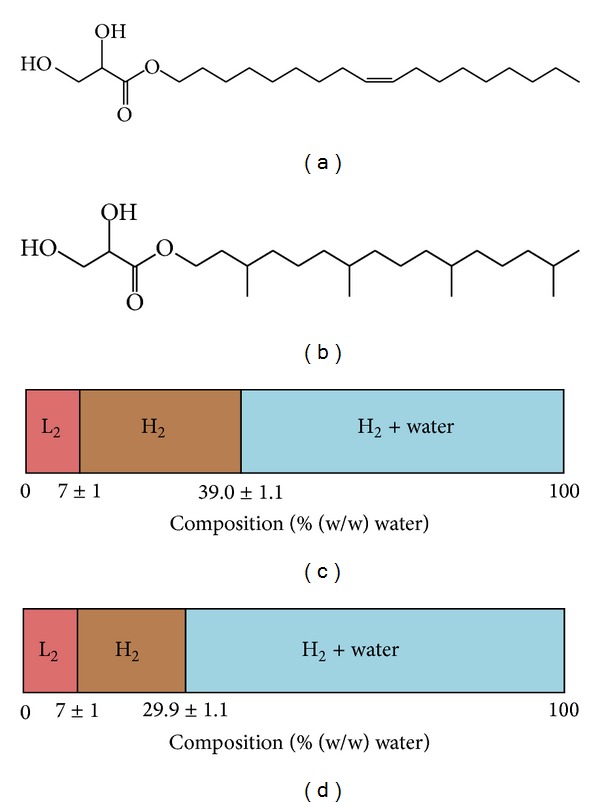
Chemical structures of (a) OG and (b) PG and one-dimensional phase diagrams for (c) OG and (d) PG at 37°C showing water content at phase boundaries for reverse micellar (L_2_) and reverse hexagonal phase (H_2_) (adapted from [10, 50]).

Oleyl glycerate (OG, 2,3-dihydroxypropionic acid octadec-9-enyl ester) and phytanyl glycerate (PG, 2,3-dihydroxypropionic acid 3,7,11,15-tetramethyl-hexadecyl ester) are found to form hexagonal phase at physiological temperature when exposed to excess water, which further expands the lipid pool to form hexagonal phases [53–55]. The molecular structures and phase behaviors of OG and PG are presented in Figure 3. Boyd et al. [10] reported that a series of model hydrophobic and hydrophilic drugs, such as paclitaxel, irinotecan, glucose, histidine, and octreotide, can be incorporated into OG- and PG-based hexagonal phases and in vitro drug release was shown to follow the Higuchi diffusion controlled release profile. Although the understanding of these new glycerate esters is limited, for example, their long-term safety, tolerability, the susceptibility to esterases, and local toxicity of their degradation products, they are promising materials due to their lower melting point and improved thermal stability compared to GMO [10].

## 3. The Influencing Factors for the Phase Transition of Cubic and Hexagonal Liquid Crystals

Many factors influence the phase behaviors of cubic and hexagonal liquid crystals, such as the molecular structures of lipids, pressure, temperature, salt concentration, pH, and addition of a third substance. Full understanding of these influencing factors on the phase transition will provide better quality control as well as expanding the lipid applications in drug development.

The molecular structures of lipids play an important role in the determination of phase behavior. The critical packing parameter (*P*) is used to predict the nanostructure of formed liquid crystal with the formula *P* = *v*/*al*, where *P* is critical packing parameter, *v* is the hydrophobic chain volume, *a* is the cross-sectional area of the polar headgroup, and *l* is the hydrophobic chain length [56]. It is important to note that the *P* value (and therefore the self-assembled nanostructure) will change along with other parameters such as temperature and solvent conditions. Depending on *P*, different self-assembled liquid crystalline structures will be formed [17]. When *P* = 1, lamellar liquid crystalline structure forms. When *P* < 1, oil-in-water self-assembled structures form, such as normal micelles (L_1_), normal cubic structure (V_1_), and normal hexagonal phases (H_1_). When *P* > 1, water-in-oil self-assembled structures form, such as reversed micelles (L_2_), reversed cubic structure (V_2_), and reversed hexagonal structure (H_2_).

Yaghmur et al. [57] investigated the impact of pressure and temperature on the stability of the inverted type discontinuous cubic phase (Fd3m) versus the inverted type hexagonal phase (H_2_). The results showed that compressing the Fd3m phase under ambient temperature induced the transition of Fd3m to H_2_. Interestingly, the temperature-dependent structural transition demonstrated the opposite trend where an increase of temperature induced the structural transition from H_2_ to Fd3m at isobaric condition.

Salt concentration [58–60] and pH value [61–63] also have an impact on the phase behavior of liquid crystals to a certain extent. For example, Awad et al. [58] found out that low concentrations of Ca^2+^ induced the L_*α*_ phase to the cubic phase transition in the suspensions of monoolein and dioleoylphosphatidylglycerol mixtures. Sallam et al. [64] observed that the reversed hexagonal phase occurred at a lower temperature in simulated gastric fluid than that in simulated intestinal fluid or water, which indicated that the formation of hexagonal phase was favored in an acidic environment. In addition, Negrini and Mezzenga [29] suggested that a system composed of monolinolein and linoleic acids (97/3, w/w) was able to reversibly change from a reverse bicontinuous cubic phase to a reverse hexagonal phase, when the pH was dropped from 7 to 2. Yaghmur et al. [65, 66] investigated the pH effect on the phase structures of 6% bupivacaine-loaded GMO-based liquid crystals, and it was found out that the different self-assembled structures were formed by increasing the pH from 6.0 to 7.4, which induced the structural transition of Pn3m + H_2_ *⟶* H_2_.

In addition, additives will modulate the textures of liquid crystals which result in phase transition. Yaghmur et al. [30] introduced tetradecane (TC) to the monolinolein- (MLO-) water-poloxamer 407 (F127) system and found out that, when mass of TC/mass of MLO = 19, the system transformed from cubosomes to hexosomes and, when mass of TC/mass of MLO = 75, the system transformed from hexosomes to a concentrated microemulsion. Later on they [67] found out that diglycerol monooleate or soybean phosphatidylcholine had a countereffect on that of tetradecane which turned back the self-assembled nanostructures in the TC-loaded dispersions from hexosomes to cubosomes. Shah and Paradkar [68] studied the effect of the additives with different hydrophilic-lipophilic balances (HLBs of 1.5, 3, 4, 5, 7, 10, and 11) in GMO matrices on their phase transformation. The results showed that the GMO matrices incorporated with lower HLB additives (HLBs of 1.5, 3, 4, and 5) formed hexagonal phase after 12 h of hydration. This may be because the incorporated hydrophobic additives were dissolved in lipophilic domain of GMO, thus increasing the apparent hydrophobic chain volume of the lipid. Therefore, it might increase the *P* value of GMO and transform mesophase from cubic phase to hexagonal phase. Interestingly, the GMO matrices incorporated with higher HLB additives (HLBs of 7, 10, and 11) formed lamellar phase after 12 h of hydration. The authors proposed that these hydrophilic additives with higher HLB reduced the access of water to GMO and thus caused such transformation. Amar-Yuli and Garti [69] investigated the impact of incorporation of triglycerides (TAGs) with various chain lengths into the binary GMO/water system, and the results suggested that the solubilization of TAG in the system facilitates transition from lamellar or cubic phases to hexagonal structure at room temperature. Among all the tested TAGs, tricaprylin in the binary GMO/water system provided direct transformation of the lamellar phase to the hexagonal phase, but without the formation of a cubic structure. They suggested that TAG between GMO tails would change the geometry of monoolein molecules from cylindrical to wedge shape and thereby caused the phase transition. Dong et al. [46] found out that the presence of 5% w/w vitamin E acetate in the PT-water system suppressed the transition temperature of the cubic phase to hexagonal phase from 60°C to below 25°C. This indicated that lipophilic components in liquid crystalline formulations, although they account to relatively small amount, may have a significant impact on the phase behavior.

## 4. Applications of Cubic and Hexagonal Liquid Crystals as Drug Delivery Systems

Since the advent of the first patent in 1984 [45] when the highly ordered cubic phase was proposed as an interesting matrix in controlled release preparations, lipid-based liquid crystal systems have been extensively investigated in drug delivery, as well as the potential application in theranostic nanomedicines [70–72]. In this section, some applications of cubic and hexagonal liquid crystals as drug carriers are reviewed. Tables 1 and 2 list some such drug delivery systems in recent years (2007–2012).

### 4.1. Cubic Liquid Crystals as Drug Delivery System

#### 4.1.1. Ability to Sustain or Control Drug Release

As drug carriers, cubic phase liquid crystals have the ability to provide sustained drug release. Drugs with a wide range of molecular weights and water solubilities have demonstrated sustained release in a cubic phase, such as aspirin and vitamin E [82], propantheline bromide and oxybutynin hydrochloride [83], metronidazole [84], tetracycline [85], timolol maleate [86], chlorpheniramine maleate [87], propranolol hydrochloride [88], melatonin, pindolol, propranolol and pyrimethamine [89], hemoglobin [90], cefazolin [91], insulin [92, 93], capsaicin [26], cinnarizine [51, 94], and diclofenac salts [95]. Lee et al. [73] studied the in vitro sustained release behavior of a number of model hydrophilic drugs with various molecular weights (^14^C-glucose, Allura Red, and fluorescein isothiocyanate dextrans FD-4, FD-20, and FD-70) in two types of liquid crystalline matrixes, namely, V_2GMO_ (a cubic phase prepared from GMO) and V_2PT_ (a cubic phase prepared from PT). The release samples were constrained in microbeakers with a fixed surface area to ensure a constant release area between the liquid crystals and the release media. The results showed that in all cases the cumulative amount of drug diffusion through the matrix followed a linear relationship with the square root of time, which represented a Higuchi diffusion controlled release profile. The influence of phase structure and molecular weight on drug release was also investigated. It was discovered that the release rate of each drug decreased as the matrix was changed from V_2GMO_ to V_2PT_ and the diffusion coefficient of the model drugs was reduced as the molecular weight increased. The results indicated that phase type and molecular weight of drugs had an influence on their release behavior. In order to confirm whether the in vitro release data were able to translate into in vivo oral absorption dates, the oral absorption kinetics of ^14^C-glucose formulations were further studied in rats. In this study, ^14^C-glucose was chosen as the model drug due to its fast absorption rate; therefore, the kinetics of ^14^C-glucose in plasma would only be determined by its release rate from the matrix. The pharmacokinetic results showed that the mean* t*_max_ (time to peak) of ^14^C-glucose absorption in solution formulations (1.00 h) was the shortest, followed by V_2GMO_ (1.13 h) and V_2PT_ (1.88 h). It was found out that the oral absorption rate of ^14^C-glucose followed the order observed in vitro, which indicated that the nanostructure of these lipid-based liquid crystalline systems had a significant impact on oral absorption of hydrophilic drugs.

Recently, Nguyen et al. [51] firstly demonstrated the sustained absorption of a poorly water-soluble drug (cinnarizine) when incorporated into PT-based cubosomes after oral administration. The plasma profiles showed that the plasma concentration of PT-based cubosomes was maintained at 21.5 ± 1.5 ng/mL within 12 and 48 h, while the plasma concentrations dropped to below quantification limit after 24 h when treated with suspensions or GMO-based cubosomes. They further investigated the mechanism of PT- and GMO-based cubosomes upon oral administration. The small-angle X-ray scattering results showed that the nanostructure of PT-based cubosomes was well maintained over 18 h in both simulated gastric and intestinal fluids. In contrast, the cubic phase structure of GMO-based cubosomes was collapsed within 2 h in simulated intestinal fluid or 18 h in simulated gastric fluid. This suggested that degradation of the nanostructure lead to fast drug release. In addition, in gastrointestinal retention studies, it was found out that over 10% of given PT and cinnarizine remained in stomach at 24 h after oral administration. In sharp contrast, GMO and cinnarizine were below quantification levels after 4 h. Therefore, the nondigestible nature of PT and the maintenance of the cubosome structure contributed to the sustained release and absorption of cinnarizine in stomach.

Esposito et al. [96] studied the performance of cubosomes as sustained percutaneous delivery systems with the model drug molecule of indomethacin. The in vitro diffusion study showed that the flux of indomethacin from carbomer-indomethacin loaded cubosomes (A) was significantly lower than carbomer-blank cubosomes spiked with free indomethacin (B) and carbomer with an indomethacin water suspension (C). Furthermore, in vivo results indicated that formulation A significantly prolonged the release of indomethacin thus exhibiting a significant long-lasting anti-inflammatory activity when exposed to UVB (ultraviolet-B) irradiation for 6 h, whereas the anti-inflammatory effect of the other two formulations was time-limited. The results confirmed that the main factor controlling drug diffusion is the nanostructure of cubosomes rather than the viscosity of the carbomer gel. In addition, tape-stripping experiment was conducted to understand the drug release mechanism, and it was found out that the amount of indomethacin recovered in the stratum corneum after the removal of formulation A was remarkably higher than that in the other two formulations. Notably, the amount of indomethacin recovered from the removal of formulations B and C was not significantly different. The authors proposed that the cubosomal GMO interacted with the stratum corneum lipids and it might lead to the formation of a cubosomes depot in stratum corneum from which indomethacin was released in a controlled fashion.

#### 4.1.2. Ability to Improve Drug Bioavailability and Reduce Drug Toxicity

Cubic phases are used to improve the drug bioavailability and reduce drug toxicity. Yang et al. [74] prepared PT-based cubosomes containing amphotericin B (AmB) to improve its bioavailability and reduce nephrotoxicity. After oral administration of an AmB-loaded cubosomal formulation in rats, nephrotoxicity was not observed and the relative bioavailability of AmB was approximately 285% compared to the control group. Chen et al. [77] developed a cyclosporine A-loaded GMO/F127 cubosome system to reduce ocular irritancy. The results showed that cubosomes almost had no irritation, and only transient corneal hyperemia was observed in one rabbit but recovered within 1 h. In addition, no ocular damage or clinically abnormal signs were noticed in cornea, conjunctiva, or iris.

#### 4.1.3. Ability to Enhance the Stability of Drugs

The unique structure of the cubic phase is used to incorporate unstable drug substances and protect them from physical and chemical degradation. Sadhale and Shah [92, 93] studied the ability of cubic phase gel to protect insulin from agitation-induced aggregation. The results showed that the native conformation of agitated insulin in cubic phase gels was almost unaffected for 2 months at 37°C, while the majority of insulin in solution appeared to aggregate and precipitate only after 8 days. Therefore, the cubic phase gel was able to protect insulin from agitation-induced aggregation and subsequent precipitation. Furthermore, they [93] investigated the effect of agitation on biological activity of insulin in cubic phase gel by subcutaneous injections of the agitated cubic phase gel, nonagitated cubic phase gel, agitated insulin solution, and normal saline to fasted rats and their blood glucose levels were measured. The blood glucose levels given the nonagitated cubic phase gel and the agitated cubic phase gel were significantly lower (*P* < 0.05) than those in the agitated insulin solution or saline from 40 min to 4 h. The results suggested that insulin was biologically active in both agitated and nonagitated cubic phase gels. However, upon agitation, insulin in solution totally lost its hypoglycemic activity. In summary, GMO-based cubic phase gel can protect insulin from agitation-induced aggregation. Sadhale and Shah [91] also evaluated the stability of two model drugs, cefazolin and cefuroxime, in a GMO cubic phase gel. The stability of cefazolin was assessed at two different concentrations (200 and 50 *μ*g/g), at 22 and 37°C. The results showed that the degradation of cefazolin at lower concentration was 3- and 18-fold slower in cubic phase gel than that in solutions at 22 and 37°C, respectively. At 22 and 37°C, the kinetics of degradation at higher concentration of cefazolin was not first-order but a lag phase followed by an exponential loss of cefazolin, which may be due to its oxidation. Later on, the oxidation of cefazolin was confirmed by its 18-fold higher stability in the presence of EDTA and nitrogen in solution. In addition, the degradation rate of cefuroxime was 2 times slower in cubic phase gel than that in solution. In summary, this study clearly demonstrated that cubic phase gel enhanced the chemical stability of cefazolin and cefuroxime.

#### 4.1.4. Ability to Increase the Penetration of Drugs

Cubic phases and cubosomes also have the ability to improve the transdermal/topical delivery of small molecules such as acyclovir [97], paeonol [75], *δ*-aminolevulinic acid [98], sulphorhodamine B [76], calcein [99], and diclofenac salts [95], as well as macromolecules such as cyclosporin A [77, 100]. Helledi and Schubert [97] reported that the cubic phase enhanced the penetration of acyclovir by 6 times compared to its commercial product. Luo et al. [75] prepared a cubic gel containing 3% paeonol, 30% water, and 67% GMO, and the in vitro skin permeability test showed that the permeability coefficient of cubic gel was 8.34 ± 0.49 × 10^−3^ cm/hour, which was significantly higher (*P* < 0.05) compared to the microemulsion gel (5.88 ± 0.28 × 10^−3^ cm/hour) and the control solution (3.06 ± 0.10 × 10^−3^ cm/hour). The results indicated that paeonol in cubic gel had higher permeability than that of paeonol in microemulsion gel and the control solution; therefore, cubic gel would be a better carrier for paeonol in transdermal delivery. Lopes et al. [100] reported that the cubic phase increased the penetration of cyclosporin A in stratum corneum and epidermis plus dermis ([E + D]) at 12 h postapplication in vitro. The in vivo penetration study showed that the cubic phase increased the concentration of cyclosporin A in the stratum corneum and [E + D] by 2.5 and 2 times, respectively, when compared to the control formulation. Chen et al. [77] prepared a cyclosporine A-loaded cubosome system for ocular drug delivery to improve corneal penetration. The in vitro corneal penetration study showed that the steady-state permeation rate of cyclosporine A was 1.52-fold compared to that of the oil solution. In addition, cyclosporine A-loaded cubosomes demonstrated a significantly enhanced initial penetration rate, where the cumulative amount of cyclosporine A in the first 0.5 h was about 2-fold compared to that of the oil solution. The authors concluded that the main mechanism of the enhanced corneal permeation might be the absorption and/or surface lipid exchange between the liquid crystalline nanoparticles and corneal epithelial cells. In addition, the close adhesion of the small lipid colloidal carriers with a large surface area to the lipophilic epithelium surface could promote a quick initial permeation rate. It was also reported that the periodically curved lipid bilayer of liquid crystalline nanoparticles was very similar to the microstructure of the cell membrane [101, 102] and the cubic architecture in stratum corneum was quite similar to the structure of cubic phases [103]. Furthermore, the interaction between biological tissues and cubic phases may contribute fast permeation rate as well, and this property makes the development of cubosome-based products more promising.

Recently, some advanced optical technologies have been used to investigate the penetration mechanism of fluorescent materials. The method of fluorescent quantification at the skin surface was used to quantify the penetration of *δ*-aminolevulinic acid (ALA) and its methyl ester into the tissue [98]. ALA is a precursor of heme which can induce the production of the photosensitizer protoporphyrin IX (PpIX) in living tissues. A fiber-optic probe coupled to a spectrophotometer was used to measure the PpIX fluorescence at the skin surface after topical administration of cubic phases. The results showed that, after 1 h application, GMO-based cubic systems and PT/propylene glycol/water cubic systems showed significantly higher fluorescence than the standard ointment over 10 h (*P* < 0.05), and this was probably due to the enhanced drug penetration. The authors also analyzed the difference between GMO and PT cubic systems in terms of enhanced drug permeation and concluded that the difference mainly relied on the swelling extent and the rheological property of the systems. Notably, the maximum amount of water in the PT-based cubic phase (about 28%) was less than that in GMO-based cubic phase (about 40%), which implied that PT-based cubic phase had more narrow water channels thus resulting in a slower release. In terms of rheological property, PT-based cubic phase had higher viscosity which made it quite difficult to handle and contact with skin thereby led to a lower PpIX level. In addition, propylene glycol was introduced into PT-based cubic phase to make the system softer, and this may be a partial explanation for the higher PpIX level in the PT-based cubic phase with propylene glycol. In another study, two-photon microscopy [76] was used to visualize the uptake of sulphorhodamine B (incorporated into GMO-/PT-based cubic phases) in full-thickness human skin. This technology is able to take images of fluorophores of sulphorhodamine B much deeper into highly light-scattering and light-absorbing tissues compared to confocal laser scanning microscopy, but with minimal photobleaching and phototoxic effects. The results suggested that the dominating delivery route of the cubic phases is via microfissures caused by microscopic clustering of the keratinocytes in the skin. Therefore, sulphorhodamine B delivered by cubic phases diffused into the surrounding intercellular lipid matrix through these microfissures and acted like a source for its sustained release.

### 4.2. Hexagonal Liquid Crystals as Drug Delivery System

#### 4.2.1. Application of OG-Based and PG-Based Hexagonal Phase

Due to the limit of materials and preparation conditions, hexagonal liquid crystals as drug carriers are less reported than those of cubic liquid crystals. Fortunately, in recent years, Boyd et al. [10] synthesized a new class of materials with glycerate headgroups to form lyotropic liquid crystal. Among these materials, OG and PG were found to form hexagonal phase in excess water at physiological temperature. Thereafter, the OG- and PG-based hexagonal phase matrices were applied as the carriers for a series of model hydrophobic and hydrophilic drugs, such as paclitaxel, irinotecan, glucose, histidine, and octreotide, and the in vitro release studies showed that in all cases their release behavior obeyed Higuchi kinetics. This suggested that OG- and PG-based hexagonal phases have the ability to provide sustained drug release.

In another study, Boyd et al. [78] reported that the OG-based hexagonal phase improved the oral bioavailability of cinnarizine up to 3.5 and 3 times than that of the control suspension and GMO-based cubic phase, respectively. The in vitro lipolysis experiments showed that all of the GMO had been digested within the first 5 min. In contrast, although the hydrolysis of OG was also fast in the first 5 min, it kept steady in the following 55 min. It suggested that OG was less susceptible to hydrolysis by pancreatic lipase than GMO and this might be responsible for increased oral bioavailability of cinnarizine when incorporated into OG formulations.

OG-based hexosomes were also used to improve the stability of irinotecan [50]. Irinotecan is a highly effective anticancer drug which displays a pH dependent equilibrium between its active lactone and inactive carboxylate forms, with rapid conversion to the carboxylate form occurring at a neutral pH. The stability studies showed that over 53-day storage of irinotecan-loaded hexosomes at 40°C, the lactone : carboxylate ratio increased from the initial value of 94 : 6 to 100 : 0, and 4% of the OG was hydrolysed to glyceric acid which led to the reduction of pH of the system from 6.6 to 3.5. The decreased pH may favor the equilibrium towards the lactone form of irinotecan. The results suggested that OG-based hexosomes improved the retention of irinotecan as the lactone form and might be a promising alternative to current irinotecan formulations.

#### 4.2.2. Ability to Increase the Penetration of Drugs

Hexagonal phases and hexosomes also have the ability to enhance the penetration of incorporated drugs. Lopes et al. [79] reported that, when incorporated into GMO-based hexagonal phase gel (GMO/vitamin K/water at 77.5/2.5/20, w/w/w) or GMO-based hexosomes (GMO/vitamin K/F127/water at 15/2.5/0.9/81.6, w/w/w/w), vitamin K was delivered to stratum corneum and [E + D] was increased to 2–3.7 times than that in a vaseline control solution. In another article, Lopes et al. [104] evaluated the ability of GMO-based hexosomes (GMO/oleic acid/F127/water at 8/2/0.9/89.1, w/w/w/w) to improve the skin penetration of a model peptide (cyclosporin A) both in vitro and in vivo. An in vitro skin penetration study showed that the maximal concentrations of cyclosporin A in the stratum corneum and in the [E + D] were 2-fold higher when incorporated into hexosomes than the control formulation (olive oil). Similarly, in vivo, the concentration of cyclosporin A-loaded hexosomes was 1.5 and 2.8 times higher than that in the stratum corneum and [E + D], respectively. The results suggested that hexagonal phase enhanced the cutaneous penetration of both vitamin K and cyclosporin A. Recently, Penetratin, a cell penetrating peptide which was embedded within the hexagonal phase liquid crystal, was used to improve the transdermal delivery of incorporated sodium diclofenac [80, 81]. An in vitro skin penetration study revealed that the permeability coefficient of sodium diclofenac enhanced 2.2 times in the presence of 1.4 wt% Penetratin. The mechanism of Penetratin-enhanced sodium diclofenac permeation through porcine skin in vitro was studied with an attenuated total reflectance Fourier transform infrared (ATR-FTIR) technique. ATR-FTIR skin studies implied that Penetratin accelerated the structural transition of skin lipids from a hexagonal symmetry to a liquid-disordered state. This structural change resulted in faster diffusion of sodium diclofenac through the stratum corneum. Swarnakar et al. [14] prepared a progesterone-loaded hexosome system for mucosal delivery and investigated the possible penetration mechanisms of the carriers. After the application of progesterone-loaded hexosomes to the excised rabbit buccal mucosa for 12 h, the observed flux was 5 times higher than that of a progesterone-loaded gel and nearly 4 times higher than a plain progesterone suspension. Fourier transform infrared spectrum and confocal laser scanning microscopy of treated mucosa showed lipid extraction phenomena as well as evident pores, indicating a probable intercellular “virtual channel” available to facilitate the penetration of hexosomes.

#### 4.2.3. Application in Stimuli Responsive Drug Delivery System

Because of the possibility to switch between the cubic phase (fast release) and hexagonal phase (slow release) by adjusting temperature and/or pH, researchers have designed a series of stimuli responsive drug delivery systems. Fong et al. [105] developed an externally regulated thermoresponsive liquid crystalline system for subcutaneous injection of hydrophilic drugs (glucose). In in vivo absorption studies, the drug was released slowly from hexagonal phase when subcutaneously injected at physiological temperature. After application of a cool pack at the injection site, the plasma concentration was significantly increased due to the structure transformed into cubic phase. The authors suggested that this system would be a suitable carrier for the delivery of short-acting hormones. Negrini and Mezzenga [29] developed a food-grade lyotropic liquid crystal system, which was composed of monolinolein and linoleic acids (97/3, w/w) in the presence of excess water. The system was able to reversibly convert from cubic phase to hexagonal phase, when changing the pH from neutral (pH 7) to acidic (pH 2) conditions. In vitro drug release and diffusion experiments showed that drug release rate was 4 times faster in cubic phase at pH 7 (intestine) than the hexagonal phase at pH 2 (stomach), which suggested that this system may be an ideal candidate for intestine- or colon-targeted delivery.

## 5. Conclusion

This review mainly discusses the investigation of current status of lipid-based liquid crystalline phases as drug delivery systems. Although the cubic and hexagonal phases have been extensively investigated as potential sustained release systems for nearly 30 years, more work needs to be done to further evaluate them in drug delivery systems. Studies with respect to the interactions between the drug and the liquid crystalline phases in vivo are very limited, and the impact of the biological environment on drug release kinetics and biodegradation is also not well understood. The commercialization of these systems in clinical application is challenging, primarily due to the lack of a suitable, scalable manufacturing method. In addition, the choice of commercially available lipids with a desired phase behavior is limited. Nevertheless, the future of lipid-based liquid crystalline delivery systems remains exciting in both academic and industrial sectors.

## Figures and Tables

**Table 1:** Cubic phases as drug carriers in recent years (2007–2012).

Type of cubic phases	Composition	Bioactive molecules	Administration route	Reference
Cubic phase gel	GMO/water and PT/water	14C-glucose, Allura Red, and fluorescein isothiocyanate dextran	Oral	[73]
Cubosomes	GMO/F127/water and PT/F127/water	Cinnarizine	Oral	[51]
Cubosomes	PT/F127/water	Amphotericin B	Oral	[74]
Cubic phase gel	GMO/propylene glycol/water	Capsaicin	Transdermal	[26]
Cubic phase gel	GMO/water	Paeonol	Transdermal	[75]
Cubic phase gel	GMO/water and PT/water	Sulphorhodamine B	Transdermal	[76]
Cubosomes	GMO/F127/water	Cyclosporine A	Ocular	[77]

**Table 2:** Hexagonal phases as drug carriers in recent years (2007–2012).

Type of cubic phases	Composition	Bioactive molecules	Administration route	Reference
Hexagonal phase gel	OG/water	Cinnarizine	Oral	[78]
Hexagonal phase gel	GMO/vitamin K/water	Vitamin K	Transdermal	[79]
Hexosomes	GMO/vitamin K/F127/water	Vitamin K	Transdermal	[79]
Hexagonal phase gel	GMO/TAG/water	Sodium diclofenac	Transdermal	[80, 81]
Hexosomes	GMO/oleic acid/F68/water	Progesterone	Oromucosal	[14]
